# Favorable clinical outcomes of checkpoint inhibitor-based combinations after progression with immunotherapy in advanced non-small cell lung cancer

**DOI:** 10.20517/cdr.2021.28

**Published:** 2021-05-24

**Authors:** Xin Yu, Xiangling Chu, Yan Wu, Juan Zhou, Jing Zhao, Fei Zhou, Chaonan Han, Chunxia Su

**Affiliations:** Department of Oncology, Shanghai Pulmonary Hospital & Thoracic Cancer Institute, Tongji University School of Medicine, Shanghai 200433, China.; ^#^Authors contributed equally.

**Keywords:** Immunotherapy, non-small cell lung cancer (NSCLC), patterns of progression, treatment beyond progression (TBP)

## Abstract

**Aim: **Immune checkpoint inhibitors (ICIs) have dramatically changed the treatment paradigm in patients with non-small-cell lung cancer (NSCLC). However, progression patterns with immunotherapy are currently unclear and therapeutic options beyond resistance remain challenging.

**Methods: **We reviewed advanced NSCLC patients between January 2016 and December 2019 who were treated with anti-PD-1/PD-L1 inhibitors in our center and identified those who developed disease progression. Later-line treatment strategies were collected and objective response rate, progression-free survival (PFS), and overall survival (OS) were assessed.

**Results: **Of the 118 patients, 46 (39.0%) showed oligoprogression and 72 (61.0%) showed systemic progression. No difference in progression patterns was observed between monotherapy and combination therapy. Systemic progression was strongly associated with never-smokers (51.4% *vs.* 21.7%, *P *= 0.001) and ECOG PS = 2 (13.9% *vs.* 2.2%, *P *= 0.048) at baseline. The distribution of progression sites was roughly similar between oligoprogression and systemic progression, and the most commonly affected anatomic site was lung (66.9%), followed by bone (12.7%) and lymph nodes (11.0%). For patients beyond first disease progression, checkpoint inhibitor-based combinations could lead to a significantly longer PFS2 compared with ICIs monotherapy (9.63 months *vs.* 4.23 months, *P *= 0.004, HR = 0.394, 95%CI: 0.174-0.893) and other therapy (9.63 months *vs.* 4.07 months, *P *= 0.046, HR = 0.565, 95%CI: 0.326-0.980). Median OS of the ICIs combination group was not reached but was significantly longer than other therapy group (NR *vs.* 14.37 months, *P *= 0.010, HR = 0.332, 95%CI: 0.167-0.661).

**Conclusion: **Systemic progression occurs more frequently among NSCLC patients receiving ICIs. Checkpoint inhibitor-based combinations show favorable outcomes as subsequent treatment strategies after the failure of previous ICIs treatment.

## INTRODUCTION

Advances in immune checkpoint inhibitors (ICIs) targeting programmed cell death protein-1 (PD-1) and its ligand programmed cell death ligand-1 (PD-L1) have dramatically changed the treatment landscape for non-small cell lung cancer (NSCLC), providing durable clinical response in a subset of patients^[1,2]^. Long-term responses in advanced stage disease have been accomplished with a 5-year overall survival (OS) of 20% in unselected population and up to 40% in patients with high PD-L1 expression^[3]^. However, the majority of patients treated with ICIs are either non-responders or eventually develop progressive disease (PD). The progression patterns of immunotherapy are far from completely understood. Uncertainties remain regarding the best way to manage resistance, and prospective clinical trials which demonstrate treatment strategies following progression are lacking.

Oligometastatic disease was first proposed by Hellman and Weichselbaum in 1995 as an intermediate state between locally confined and polymetastatic cancers^[4]^. Although a clear definition is not reported, oligoprogression has been proposed and described as a special clinical situation where a limited number of metastatic tumor sites have progressed. It is a relatively new concept denoting anatomically restricted tumor progression in patients. Thus, local therapies including stereotactic body radiotherapy (SBRT), ablation, and surgical operations are applied as the management of oligoprogression. This may allow the continuing use of systemic treatments and result in a prolonged response^[5]^. In contrast, systemic progression usually implies polymetastatic disease and its pathogenesis is considered to be complex, as numerous parameters including molecular evolution of cancer cells, changes of tumor microenvironment, and hemodynamic alternations could potentially be involved. The identification of patterns of failure at disease progression is of significant importance as it provides information for subsequent treatment choices. Previous studies have reported the relapse pattern in NSCLC under targeted therapy, with the occurrence of oligoprogression varying between 30% and 70%^[6-9]^. However, the progression patterns of immunotherapy and their correlations with clinical parameters remain to be explored.

Emerging data show that patients treated with ICIs might experience an atypical response pattern compared with chemotherapy and targeted therapy due to the potential of delayed responses and the rare phenomenon of pseudo-progression, eventually with tumor shrinkage after initial disease progression^[10]^. Discordance between radiographic endpoints and OS in NSCLC patients treated with anti-PD-1/PD-L1 agents was reported in several studies, suggesting that progression-free survival (PFS) and objective response rate (ORR) based on Response Evaluation Criteria in Solid Tumors version 1.1 (RECIST v1.1) do not fully capture the clinical benefit of immunotherapy^[11-13]^. These observations indicate that ICIs could have post-progression treatment effects and provide a rationale for allowing treatment beyond progression (TBP) in selected patients.

The efficacy of TBP was evaluated in NSCLC patients from clinical trials as well as real-world clinical practice^[14-17]^. Prolonged PFS and OS have been observed in the TBP group compared with those who were switched to other therapies, mostly chemotherapy, and those who received no further therapies. Nevertheless, a retrospective study from Japan indicated the absence of significant differences in OS and survival post progression (SPP) between TBP and non-TBP groups in advanced NSCLC. Univariate analyses of the two groups revealed that long OS or SPP were associated with prognostic factors such as c-reactive protein (CRP) and advanced lung cancer inflammation index (ALI), indicating better clinical characteristics of patients continuing immunotherapy^[18]^. Another study from a European cohort observed no significant difference in terms of post-progression survival between the salvage chemotherapy and pembrolizumab beyond progression groups in PD-L1 ≥ 50% advanced NSCLC patients who progress on first-line pembrolizumab^[19]^. These inconsistent results highlight the importance of reevaluating the efficacy of various subsequent treatment regimens beyond first progression with ICIs.

Therefore, in light of uncertainties of the relapse patterns under ICIs and further treatment options, we conducted this study to investigate the patterns of failure after immunotherapy and the beneficial subsequent treatment strategies in patients with advanced NSCLC.

## METHODS

### Patient information

The medical records of patients who were treated with anti-PD-1/PD-L1 therapy in Shanghai Pulmonary Hospital (Shanghai, China) between January 2016 and December 2019 were reviewed. In total, 118 patients were identified and enrolled in this study. The inclusion criteria were: (1) pathologically or cytologically confirmed NSCLC; (2) unresectable stage IIIB/IV according to the eighth edition of the TNM classification for lung cancer; (3) received at least one cycle of ICIs as monotherapy or combination therapy in various treatment lines; (4) at least one measurable lesion according to RECIST v1.1; (5) confirmed PD using radiological examinations including chest computed tomography (CT), positron emission tomography (PET), brain magnetic resonance imaging (MRI), bone scan, ultrasound examination, or CT of the abdomen; (6) Eastern Cooperative Oncology Group performance status (ECOG PS) score of 0-2; and (7) sufficient organ function. The exclusion criteria were: (1) EGFR mutations or ALK/ROS1 rearrangements detected by amplification refractory mutation system polymerase chain reaction (ARMS-PCR) or next generation sequencing (NGS); (2) incomplete radiological records and images at baseline or at disease progression; or (3) treatment interruption for intolerable toxicity or non-medication reasons. Cases with disease progression suitable for inclusion and exclusion criteria in this study were identified by a retrospective review of radiological images to characterize the progression patterns by experienced thoracic oncologists. Here, oligoprogression was defined as localized treatment failure at one or two anatomic sites, with 1-5 progressive measurable lesions (RECIST v1.1), either new or with ≥ 20% growth of their longest diameter (short-axis in lymph nodes). Systemic progression was defined as more than five progressive measurable lesions or progression at more than two anatomic sites. Growth of non-measurable lesions was also classified as systemic progression^[20]^.

After progression with immunotherapy, later-line treatment regimens for patients were collected by medical records. Patients who had received at least one complete cycle of any treatment following failure of immunotherapy were considered evaluable and patients who were dead or lost to follow-up beyond progression were excluded from analysis. Eventually, 83 out of 118 patients remained and were divided into ICIs monotherapy group, ICIs combination group, and other therapy group. ICIs monotherapy referred to PD-1/PD-L1 inhibitors with no additional treatment. ICIs combination therapy referred to PD-1/PD-L1 inhibitors combined with chemotherapy, anti-angiogenic therapy, local therapy (including radiotherapy and surgery), or any two of them. Other therapy referred to any anticancer treatment except for PD-1/PD-L1 inhibitors, either as monotherapy or in combination with other drugs. All patients received ICIs for the first time according to the guideline. For patients who continued ICIs beyond RECIST v1.1 progression as off-label use, criteria were clinicians-assessed potential clinical benefit, stable performance status, tolerance of treatment, and no need to deliver immediate intervention to treat or prevent serious complication of progression. In addition, ICIs combined with anti-angiogenesis has not been proven as a standard therapy for NSCLC. Patients who received this regimen in this study were actually in a clinical trial.

### Study design

This retrospective, observational study was conducted to explore the patterns of failure after immunotherapy and subsequent treatment strategies which patients could benefit from. Tumor response was assessed according to RECIST v1.1. First disease response assessment was performed upon the completion of two cycles of treatment or earlier if clinically indicated. The ORR analysis was based on best overall response (BOR), as defined by the rate of partial response and complete response. Disease control rate (DCR) was assessed by measuring the rate of partial response, complete response, and stable disease. PFS1 was defined as the time from the initiation of immunotherapy to progressive disease. PFS2 was defined as the time from the first progression of immunotherapy to second progression or death of any cause. OS was defined as the time from the first progression of immunotherapy to death due to any cause. Data cutoffs were December 2020, and patients with ongoing response to subsequent treatment at this time or the last follow-up date were considered to be censored. The study was approved by the institutional review board of Shanghai Pulmonary Hospital.

### Statistical analyses

Mann-Whitney *U* test was used on continuous variables and Pearson’s *χ*^2^ or Fisher’s exact test on categorical variables. PFS and OS were estimated using the Kaplan-Meier method and the log-rank test was used to assess differences between groups. A two-sided *P*-value of < 0.05 was considered statistically significant. All statistical analyses were performed using SPSS version 25.0 (Chicago, IL, USA) or GraphPad Prism version 8.0 (San Diego, CA, USA).

## RESULTS

### Patient population

**Figure 1:**
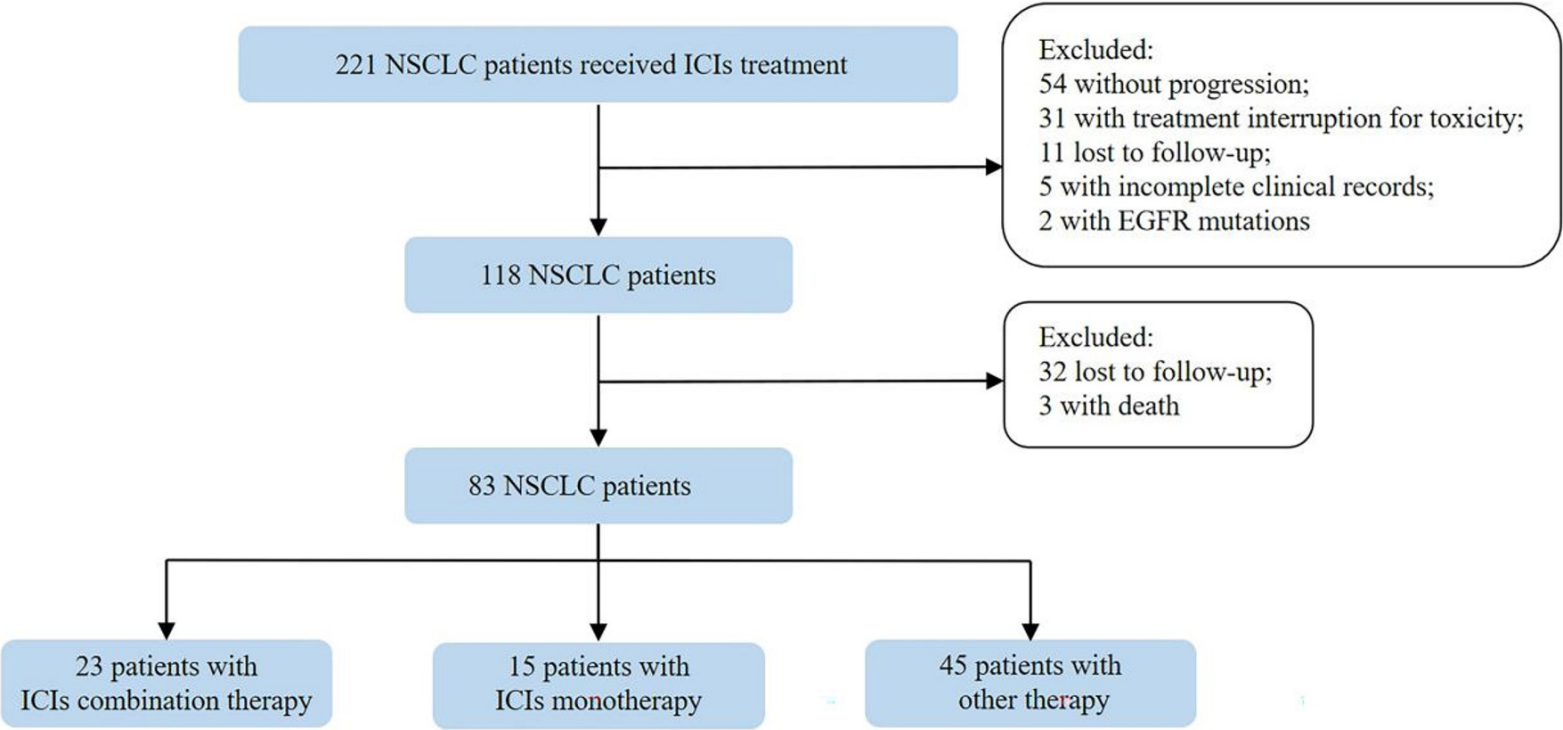
Flow chart of the study. NSCLC: Non-small cell lung cancer; ICIs: immune checkpoint inhibitors; EGFR: epidermal growth factor receptor.

Between January 2016 and December 2019, 118 patients who experienced disease progression after immunotherapy were enrolled as previously described [Figure 1]. The median age of all patients was 62 years (range, 36-80 years) and 81% were male. The predominant histology of tumors was non-squamous NSCLC (74/118, 62.7%). In total, 71 (60.2%) patients were current or former smokers. Immunotherapy was used as first-line treatment in 35 (29.7%) patients, second-line in 50 (42.4%) patients, and third-line and beyond in 33 (27.9%) patients. More patients were treated with ICIs monotherapy (68/118, 57.6%) than with chemo-immunotherapy combination (37/118, 37.4%) or immunotherapy-anti-angiogenesis combination (13/118, 11.0%). Baseline demographic and clinical characteristics of patients are summarized in Table 1.

**Table 1:** Baseline demographic and clinical characteristics of patients stratified by patterns of progression (*n *= 118)

**Characteristics**	**No. patients (%)**			
	**Total (*n* = 118)**	**Oligoprogression** **(*n* = 46)**	**Systemic progression** **(*n* = 72)**	***P*** **-value**
**Median age, year (range)**	62 (36-80)	63.5 (46-77)	60.5 (36-80)	0.250
**Gender**				0.059
Male	95 (80.5)	41 (89.1)	54 (75.0)	
Female	23 (19.5)	5 (10.9)	18 (25.0)	
**ECOG PS**				0.048
0-1	107 (90.7)	45 (97.8)	62 (86.1)	
2	11 (9.3)	1 (2.2)	10 (13.9)	
**Smoking history**				0.001
Current/Former	71 (60.2)	36 (78.3)	35 (48.6)	
Never	47 (39.8)	10 (21.7)	37 (51.4)	
**Histology**				0.602
Non-squamous	74 (62.7)	27 (58.7)	47 (65.3)	
Squamous	37 (31.4)	17 (37.0)	20 (27.8)	
NOS	7 (5.9)	2 (4.3)	5 (6.9)	
**PD-L1 status**				0.848
≥ 1%	23 (19.5)	9 (19.6)	14 (19.4)	
< 1%	13 (11.0)	6 (13.0)	7 (9.7)	
Not examined	82 (69.5)	31 (67.4)	51 (70.9)	
**Lines of therapy**				0.936
1	35 (29.7)	14 (30.4)	21 (29.2)	
2	50 (42.4)	20 (43.5)	30 (41.6)	
≥ 3	33 (27.9)	12 (26.1)	21 (29.2)	
**Number of metastatic sites**				
Mean (SD)	1.5 (1.2)	1.3 (1.1)	1.7 (1.2)	0.075
**Treatment strategy**				0.973
ICIs monotherapy	68 (57.6)	26 (56.5)	42 (58.3)	
ICIs + chemotherapy	37 (31.4)	15 (32.6)	22 (30.6)	
ICIs + anti-angiogenesis	13 (11.0)	5 (10.9)	8 (11.1)	
**Best response to treatment**				0.117
PR	29 (24.6)	15 (32.6)	14 (19.4)	
SD	61 (51.7)	24 (52.2)	37 (51.4)	
PD	28 (23.7)	7 (15.2)	21 (29.2)	
ORR (%)	24.6	32.6	19.4	0.105
DCR (%)	76.3	84.8	70.8	0.082
**Median PFS (months)**		5.70	5.17	0.491
First-line	8.60	8.64	8.43	0.755
Second-line and beyond	4.40	4.98	4.37	0.293

ECOG PS: Eastern Cooperative Oncology Group performance status; NOS: not otherwise specified; PD-L1: programmed cell death ligand 1; ICIs: immune checkpoint inhibitors; PR: partial response; SD: stable disease; PD: progressive disease; ORR: objective response rate; DCR: disease control rate; PFS: progression-free survival.

### Progression patterns and sites after ICIs failure

Among the 118 patients, 46 (39.0%) showed oligoprogression and 72 (61.0%) showed systemic progression. No differences in progression patterns were presented between patients treated with ICIs monotherapy and ICIs combination therapy. Interestingly, systemic progression was significantly associated with never-smokers (51.4% *vs.* 21.7%, *P *= 0.001) and ECOG PS = 2 (13.9% *vs.* 2.2%, *P *= 0.048) at baseline. A gender difference was also observed regarding the progression patterns. Female patients were more likely to undergo systemic progression (25.0% *vs.* 10.9%, *P *= 0.059). Besides, patients who developed oligoprogression exhibited a relatively higher response rate to immunotherapy compared with those who developed systemic progression (ORR: 32.6% *vs.* 19.4%, *P *= 0.105; DCR: 84.8% *vs.* 70.8%, *P *= 0.082, respectively). Median PFS of first-line immunotherapy seemed to be longer than second-line and beyond (8.60 months *vs.* 4.40 months, *P *= 0.071, HR = 0.702, 95%CI: 0.483-1.018) and was comparable between patients who had oligoprogression and systemic progression. Other clinical characteristics of patients between the two groups were well balanced [Table 1].

The anatomical sites of progression are shown in Supplementary Table 1. Lung (66.9%), bone (12.7%), and lymph nodes (11.0%) were affected most frequently. Other progression sites typically affected by NSCLC were also observed, such as brain (10.2%), pleura (10.2%), liver (5.9%), adrenal (3.4%), soft tissue (2.2%), and pericardium (2.2%). The distribution of progression sites was similar between the two groups except for pleura, which was classified into systemic progression by definition.

### Efficacy of subsequent therapy beyond RESIST v1.1 progression

**Figure 2:**
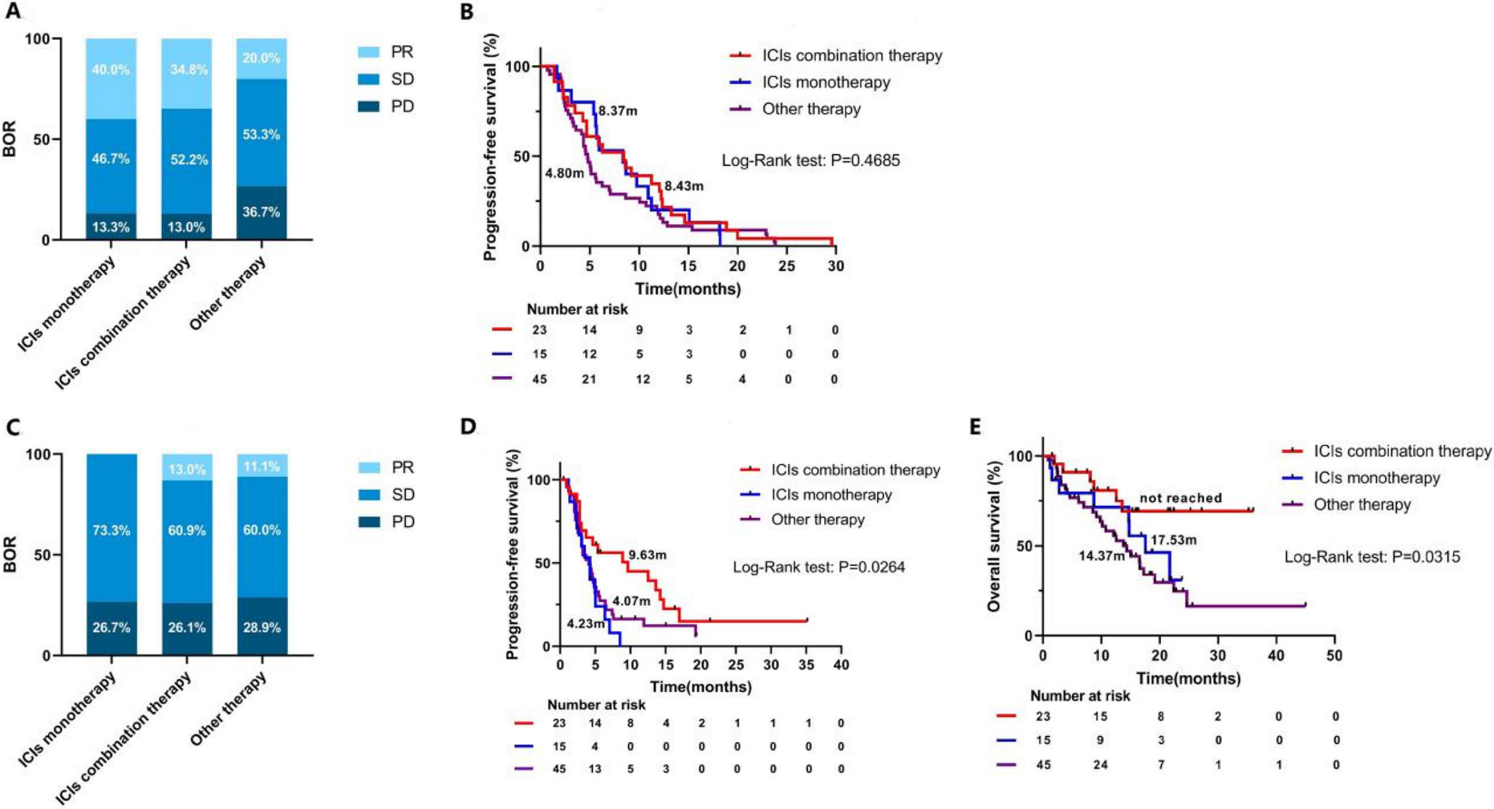
Best overall response and progression-free survival of patients stratified by treatment strategy after ICIs failure. (A) Best overall response of previous immunotherapy. (B) Progression-free survival of previous immunotherapy. (C) Best overall response of subsequent therapy. (D) Progression-free survival of subsequent therapy. (E) Overall survival of subsequent therapy. BOR: Best overall response; ICIs: immune checkpoint inhibitors; PR: partial response; SD: stable disease; PD: progressive disease.

In total, 83 patients were included in the analysis [Figure 1]. Fifteen patients (18.1%) continued the same ICIs monotherapy beyond progression as indicated by clinicians considering the possibility of pseudo-progression. Twenty-three (27.7%) received ICIs combination therapy and 45 (54.2%) switched to other anticancer therapy. Demographic and clinical characteristics beyond progression of patients are summarized in Table 2.The ICIs monotherapy group had less metastatic sites and less frequency of brain metastasis compared with the other two groups. Other clinical characteristics were balanced among the three groups. Inferior ORR and PFS1 were observed in the other therapy group compared with the ICIs monotherapy group and ICIs combination therapy group (ORR: 20.0% *vs.* 40.0%* vs.* 34.8%, PFS1: 4.80 months *vs.* 8.37 months *vs.* 8.43 months, respectively); however, the differences were not statistically significant [Figure 2A and B]. This could be explained by patients who continued immunotherapy had already experienced clinical benefit from treatment before progression. After progression with immunotherapy, the ORR of the ICIs monotherapy group, ICIs combination group, and other therapy group were 0%, 13.0%, and 11.1%, respectively (*P *= 0.365) [Figure 2C]. Surprisingly, patients who were treated with checkpoint inhibitor-based combinations had longer PFS2 compared with those treated with ICIs monotherapy (9.63 months *vs.* 4.23 months, *P *= 0.004, HR = 0.394, 95%CI: 0.174-0.893) and other therapy (9.63 months *vs.* 4.07 months, *P *= 0.046, HR = 0.565, 95%CI: 0.326-0.980). No difference of PFS2 was found between the ICIs monotherapy and other therapy groups (4.23 months *vs.* 4.07 months, *P *= 0.408, HR = 1.293, 95%CI: 0.668-2.504). In addition, OS analysis also demonstrated the superior therapeutic efficacy of ICIs combination therapy over other therapy (NR *vs.* 14.37 months, *P *= 0.010, HR = 0.332, 95%CI: 0.167-0.661) and ICIs monotherapy (NR *vs.* 17.53 months, *P *= 0.140, HR = 0.461, 95%CI: 0.157-1.356) [Figure 2D and E, Supplementary Figure 1].

**Table 2:** Baseline demographic and clinical characteristics of patients stratified by treatment strategies beyond immunotherapy resistance (*n *= 83)

**Characteristics**	**No. patients (%)**			
	**ICIs monotherapy** **(*n* = 15)**	**ICIs combination therapy** **(*n* = 23)**	**Other therapy** **(*n* = 45)**	***P*** **-value**
**Median age, y (range)**	64 (53-80)	60 (46-77)	63 (36-77)	0.258
**Gender**				0.161
Male	12 (80)	21 (91.3)	32 (71.1)	
Female	3 (20)	2 (8.7)	13 (28.9)	
**ECOG PS**				0.682
0-1	13 (86.7)	22 (95.7)	40 (88.9)	
2	2 (13.3)	1 (4.3)	5 (11.1)	
**Smoking history**				0.479
Current/Former	10 (66.7)	16 (69.6)	25 (55.6)	
Never	5 (33.3)	7 (30.4)	20 (44.4)	
**Histology**				0.253
Non-squamous	7 (46.7)	16 (69.6)	30 (66.7)	
Squamous	6 (40.0)	5 (21.7)	14 (31.1)	
NOS	2 (13.3)	2 (8.7)	1 (2.2)	
**PD-L1 status**				0.918
≥ 1%	3 (20)	5 (21.7)	8 (17.8)	
< 1%	2 (13.3)	2 (8.7)	8 (17.8)	
Not examined	10 (66.7)	16 (69.6)	29 (64.4)	
**Lines of therapy**				0.751
1	3 (20)	9 (39.1)	14 (31.1)	
2	8 (53.3)	8 (34.8)	17 (37.8)	
≥ 3	4 (26.7)	6 (26.1)	14 (31.1)	
**Number of metastatic sites**				
Mean (SD)	0.9 (0.5)	2.5 (1.9)	1.5 (1.1)	0.001
**Specific metastasis sites**				0.973
Brain	0 (0)	9 (39.1)	4 (8.9)	0.001
Liver	0 (0)	4 (17.4)	3 (6.7)	0.168
Bone	2 (13.3)	11 (47.8)	16 (35.5)	0.092
**Progression patterns of ICIs**				0.772
Oligoprogression	7 (46.7)	11 (47.8)	18(40.0)	
Systemic progression	7 (46.7)	11 (47.8)	26 (57.8)	
Not determined	1 (6.6)	1 (4.4)	1 (2.2)	
**Subsequent therapy beyond progression**				NA
PD-1/PD-L1 inhibitors	15 (100)	23 (100)	0 (0)	
Chemotherapy	0 (0)	13 (56.5)	37 (82.2)	
Anti-angiogentic therapy	0 (0)	9 (39.1)	13 (28.9)	
Local therapy	0 (0)	5 (21.7)	7 (15.6)	

ECOG PS: Eastern Cooperative Oncology Group performance status; PD-1: programmed cell death 1; NOS: not otherwise specified; PD-L1: programmed cell death ligand 1; ICIs: immune checkpoint inhibitors; NA: not applicable.

**Figure 3:**
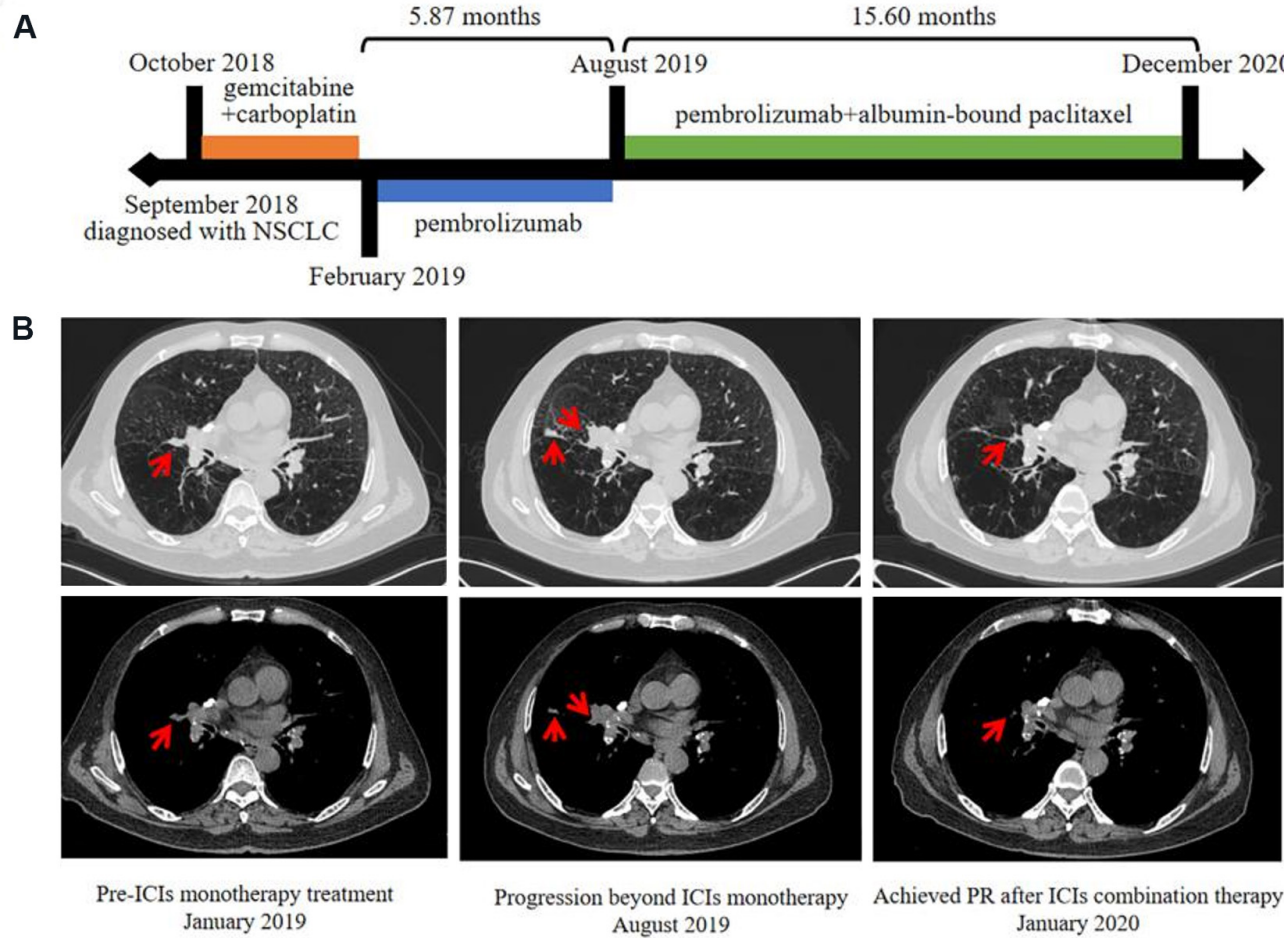
Durable clinical response to pembrolizumab plus albumin-bound paclitaxel in an NSCLC patient. (A) Clinical timeline of patient, with major treatment indicated. The patient has been benefiting from ICIs combination therapy for more than 15.60 months. (B) Chest computed tomography (CT) of the tumors before pembrolizumab initiation (January 2019), disease progression (August 2019), and achieved PR after ICIs combination therapy (October 2019). ICIs: Immune checkpoint inhibitors; NSCLC: non-small-cell lung cancer; PR: partial response.

Here, we present a typical case who benefited from chemo-immunotherapy combination beyond progression with pembrolizumab (Patient 20 in Supplementary Figure 1B). A 62-year-old male was diagnosed with wild type non-small cell lung cancer (cT3N2M1a, stage IVA) in September 2018. He was treated with gemcitabine and carboplatin as first-line therapy and discontinued after four cycles of treatment due to toxicity (Grade 4 thrombocytopenia). Pembrolizumab (200 mg Q3W) was then initiated in February 2019 until progression of the lung lesions occurred in August 2019, with stable disease as best overall response. Consequently, PFS1 for second-line immunotherapy was 5.87 months. After that, he was administered with pembrolizumab combined with albumin-bound paclitaxel and achieved PR at the first efficacy evaluation in October 2019. Until data cutoffs (December 2020), he still showed a persistent and ongoing response with checkpoint inhibitor-based combinations, resulting in a PFS2 of more than 15.60 months [Figure 3].

## DISCUSSION

The occurrence and clinical features of progression patterns in oncogene-driven NSCLC have been well characterized in several studies. Yu *et al.*^[6]^ analyzed a group of 184 EGFR patients failing first-generation TKIs and found an oligoprogression rate of 33%, 23% and 10% of whom developed intracranial and extracranial oligoprogression^[5]^. Similar trends were also discovered in other investigations, for instance, a retrospective study enrolling 266 lung adenocarcinoma patients who received first-line TKI treatment reported a lower occurrence rate of oligoprogression compared to systemic progression (37.6% *vs.* 62.4%, respectively)^[7]^. In another cohort of 206 patients who were treated with first-line EGFR-TKIs and developed oligoprogressive diseases, 124 (60.2%) cases were identified in brain^[8]^. In contrast, a higher rate of oligoprogression (73%) was observed among EGFR T790M-positive patients with acquired resistance to osimertinib, and the brain was outnumbered by other oligoprogression sites^[9]^. It could be presumed that the superior brain efficacy of osimertinib over first-generation TKIs influences the balance between intra- and extra-cranial oligoprogression.

The progression patterns under ICIs treatment have also been evaluated recently. Rheinheimer *et al.*^[20]^ observed a relatively lower rate of oligoprogression (20%) in immunotherapy-treated NSCLC and a Japanese study reported 38 out of 148 patients (26%) showed oligoprogression in their cohort^[21]^. Consistent with their results, our study demonstrated that oligoprogression occurred less frequently than systemic progression among NSCLC patients receiving ICIs, with a slightly higher rate of 39%. Besides, immunotherapy is characterized by comparable intra- and extra-cranial efficacies, which could explain the predominance of non-brain progression observed in both oligoprogression and systemic progression groups. Currently, the definition of progression patterns under immunotherapy is not completely unified and must be proposed.

Another conclusion from this study was that systemic progression was significantly associated with never-smokers and ECOG PS = 2 at baseline. In a cohort of patients with advanced NSCLC, Li *et al.*^[22]^ reported a higher ORR to immunotherapy in current and former smokers than never smokers (36% *vs.* 26% *vs.* 14%, *P *= 0.02) even when controlling for PD-L1 expression levels. Exploratory analysis also demonstrated higher 1-year survival rates in smokers compared to never-smokers^[22]^. It is hypothesized that higher mutation burden resulting from smoking improves the efficacy of immunotherapy^[23]^. Additionally, data from two Italian centers confirm poor PS was associated with reduced efficacy of immunotherapy and remained the most powerful independent prognostic factor for NSCLC^[24]^. These findings provide evidence for the potential risk of systemic progression after ICIs failure for patients who have no smoking history and poor performance status.

To date, analyses of post-progression effects of anti-PD-1/PD-L1 agents in metastatic renal cell carcinoma^[25,26]^, melanoma^[27,28]^, and NSCLC^[14,15]^ have already been reported. In the phase III OAK study, a prolonged median post-PD OS (12.7 months) was observed in atezolizumab-arm patients continuing TBP compared with patients switching to non-protocol therapy (8.8 months) and patients receiving no further therapy (2.2 months). Seven percent of the atezolizumab TBP patients achieved a post-progression response in target lesions and 49% had stable disease^[14]^. In addition, a retrospective real-world study demonstrated that a substantial proportion of NSCLC patients who were clinically stable and judged to be eligible for TBP derived a significant survival benefit from TBP^[15]^. Of note, patients in these studies received TBP with the same treatment regimen and no additional treatment modality at progression, which was known as “classical” TBP^[16]^. However, the post-progression assessment results in our study suggested that ICIs combination therapy could provide continuing anti-tumor effects and improve clinical outcomes, while ICIs monotherapy beyond first progression had shorter PFS2 and none of the 15 patients achieved PR as best response. In the context of available literature, our study is the first to provide evidence for the superior efficacy of checkpoint inhibitor-based combinations compared with ICIs monotherapy and other anticancer therapy in NSCLC patients who experienced disease progression after treatment with PD-1/PD-L1 inhibitors in clinical practice.

Rational combinations of immunotherapy with systemic therapies including chemotherapy and anti-angiogenic therapy are typically designed to impinge on distinct elements of tumor biology to achieve additive or synergistic antitumor effects. Two major ways in which chemotherapy promotes tumor immunity are by inducing immunogenic cell death as part of its intended therapeutic effect and by disrupting strategies that tumors use to evade immune recognition^[29,30]^. The combination of anti-angiogenic therapy with immunotherapy could form a reinforcing feedback loop by the reciprocal regulation between tumor vascular normalization and immune reprogramming, which remodels the tumor microenvironment and induces durable antitumor responses^[31,32]^. In addition, combining radiotherapy with immunotherapy provides an opportunity to boost the abscopal effect and extend the use of radiotherapy in both localized and metastatic disease^[33,34]^. In addition, double immune checkpoint blockades were developed with the rational of targeting complimentary pathways in tumor immune escape^[35]^. Whether continuing to boost the immune system based on ICIs in combination with other anticancer therapies after first progression is mechanistically responsible for the clinical benefit in TBP patients is unclear and remains to be clarified. In addition, it is worth noting that 6 patients in ICIs combination group (26.1%) experienced a PD as their best response to treatment, indicating a subset of patients would not benefit from ICIs combination therapy. Thus, predictive biomarkers for response such as PD-L1, TMB, soluble immunological biomarkers, and others need to be explored to optimize patient benefit^[36]^.

Our study demonstrated checkpoint inhibitor-based combinations as effective salvage therapy options for patients who have failed previous ICIs treatment with very limited therapeutic options otherwise. Decision about the discontinuation of ICIs should be cautiously considered since ICIs have shown a heterogenous spectrum of response and disease progression that may not be fully captured by conventional response criteria. Interpretation of our study must consider its retrospective nature and single-center experience. There was no controlled randomization to TBP *vs.* switching to other therapy. Thus, the criteria for TBP including clinicians-assessed potential benefit introduces bias. Besides, the baseline clinical characteristics were not well-balanced in the three groups. The ICIs monotherapy group has fewer metastatic sites and less brain metastasis. This was inevitable in a retrospective study in which clinical characteristics were not well-balanced and multivariate analysis was not applicable due to moderate sample size. Whether ICIs combination therapy showed better efficacy than ICIs monotherapy after first progression with immunotherapy still needs research. Finally, we did not discriminate and evaluate the efficacy of each treatment regimen in the combination group due to the moderate sample size, and this aspect needs to be explored. Confirmation of these findings will require prospective randomized trials in large populations.

In conclusion, the present study showed a higher frequency of systemic progression among NSCLC patients after ICIs failure and the superior efficacy of checkpoint inhibitor-based combinations as subsequent treatment strategies beyond progression.

## DECLARATIONS

### Copyright

© The Author(s) 2022.
