# Supplementary material for: Favorable clinical outcomes of checkpoint inhibitor-based combinations after progression with immunotherapy in advanced non-small cell lung cancer
Source: Cancer Drug Resist. 2021 May 24;4(3):728–39. doi: 10.20517/cdr.2021.28 (PMC9086517; doi:10.20517/cdr.2021.28)
Supplement: Supplementary file 1 [file cdr-4-728-SupplementaryMaterials.pdf]

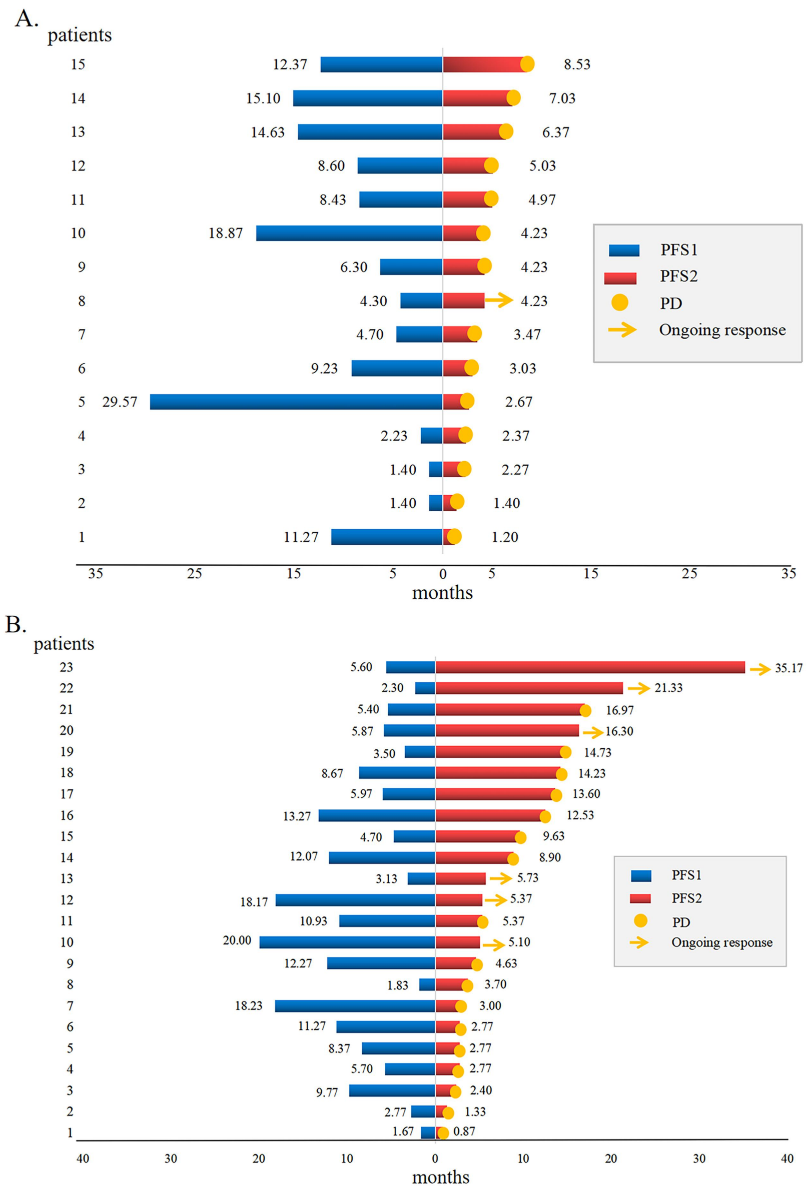

Supplementary Figure 1. Swimmers plot showing the PFS of immunotherapy and subsequent therapy. Each bar represents one patient. **A:** Patients treated with ICIs monotherapy beyond progression. **B:** Patients treated with ICIs combination therapy beyond progression. PFS: progression-free survival, PD: progressive disease.

Supplementary Table 1. Progression sites after ICIs failure

Progression sites	Total n(%)	Oligoprogression n(%)	Systemic progression n(%)	*p*-value
**Lung**	79 (66.9)	29 (63.0)	50 (69.4)	0.471
**Bone**	15 (12.7)	6 ((13.0)	9 (12.5)	0.931
**Lymph nodes**	13 (11.0)	3 (6.5)	10 (13.9)	0.213
**Brain**	12 (10.2)	6 (13.0)	6 (8.3)	0.409
**Pleura**	12 (10.2)	0(0)	12 (16.7)	0.003
**Liver**	7 (5.9)	2 (4.3)	5 (6.9)	0.560
**Adrenal**	4 (3.4)	2 (4.3)	2 (2.8)	0.646
**Soft tissue**	3 (2.5)	2 (4.3)	1 (1.4)	0.319
**Pericardium**	3 (2.5)	0(0)	3 (4.2)	0.161

ICIs: immune checkpoint inhibitors.
